# NMR-Based Fragment Screening for RNA-Targeted Drug Discovery

**DOI:** 10.3390/molecules31060916

**Published:** 2026-03-10

**Authors:** Riley J. Petersen, Yaqiang Wang

**Affiliations:** 1 Department of Biophysics, Medical College of Wisconsin, Milwaukee, WI 53226, USA; 2 Department of Obstetrics and Gynecology, Medical College of Wisconsin, Milwaukee, WI 53226, USA; 3 Medical College of Wisconsin Cancer Center, Milwaukee, WI 53226, USA

**Keywords:** RNA, NMR, fragment screening, RNA-targeted small molecule, drug discovery, RNA therapeutics

## Abstract

Fragment-based drug discovery (FBDD) has emerged as a primary approach for identifying low molecular weight leads that can be systematically optimized into high-affinity compounds. Because fragments bind inherently weakly, their detection relies on highly sensitive biophysical tools. Nuclear magnetic resonance (NMR) spectroscopy is uniquely qualified for fragment screening due to its capability in detecting weak interactions across a broad affinity range while providing site-specific binding information that supports structure-guided optimization. While FBDD is a mature field for protein targets, structured and disease-relevant RNAs have transitioned from ‘undruggable’ molecules to viable therapeutic targets for small-molecule intervention. Recent studies demonstrate that NMR-based screening can identify authentic RNA binders and guide their evolution into potent, selective ligands. This review summarizes the practical and methodological pipelines for RNA-targeted small molecule NMR screening, covering RNA construct design, sample preparation, and library pooling strategies. We evaluate both ligand- and RNA-observed NMR assays for primary hit screening and validation, integration of NMR restraints with structural modeling, and representative case studies. Finally, we discuss current bottlenecks in the field and highlight emerging strategies to accelerate the discovery of RNA-directed therapeutics.

## 1. Introduction

After decades of development, fragment-based drug discovery (FBDD) has become a mainstream strategy for identifying novel small-molecule drugs against biomolecular targets [1,2]. In contrast to high-throughput screening (HTS), which typically relies on libraries containing hundreds of thousands to millions of compounds, fragment libraries usually consist of only a few hundred to a few thousand compounds with molecular weights less than 300 Da to cover similar chemical space. Although fragment hits often exhibit weak binding affinities in the low micromolar to millimolar range, they possess high ligand efficiencies due to their small size and efficient engagement of binding sites. FBDD has gained increasing popularity due to its potential to deliver high-quality hits within a relatively short timeline and with reduced resource investment. To date, numerous fragment-derived compounds have progressed into clinical development, including seven FDA-approved drugs: asciminib [3], capivasertib [4], erdafitinib [5], pexidartinib [6], sotorasib [7], vemurafenib [8], and venetoclax [9].

A wide range of biophysics techniques are used to detect fragment binding, including surface plasmon resonance (SPR) [10], nuclear magnetic resonance (NMR) spectroscopy [11], X-ray crystallography [12], fluorescence polarization [13], and, more recently, cryo-electron microscopy (cryo-EM) [14]. NMR remains the gold standard for fragment screening because of its ability to detect weak binding interactions in solution across a broad affinity range while simultaneously providing site-specific binding interface information [15,16,17,18,19]. NMR-derived binding and orientation information can be readily integrated with computational approaches such as quantitative structure–activity relationship (SAR) analysis [20] and molecular docking to guide hit-to-lead optimization [21,22]. Consequently, this advantage ensures that NMR plays crucial roles throughout the drug discovery pipeline, including target characterization, primary screening, hit validation, hit-to-lead evolution, binding site and interface identification, and structural modeling of biomolecular complexes. NMR also provides quantitative affinity measurements through titration experiments, supports early SAR development, and can identify solvent-exposed functional groups amenable to chemical elaboration.

RNA participates in nearly all aspects of the cellular life cycle, including transcription, splicing, translation, and post-transcriptional regulation, and its dysregulation is implicated in numerous human diseases [23,24,25,26]. Despite this central biological importance, RNA has historically been underexplored as a drug target, partially because many disease-related RNA are considered undruggable due to high dynamics and the lack of distinctive ligandable pockets [27,28]. The great potential to increase the number of therapeutic targets across the entire transcriptome has encouraged growing efforts to identify small molecules against RNA [27,29,30,31,32,33,34,35,36,37,38,39]. While several RNA-targeted candidates are currently advancing through clinical development, FBDD approach has yet to produce an FDA-approved RNA therapeutic [40]. This underscores both the challenges of targeting RNA and the significant opportunity for growth as the field refines its structural and screening approaches.

While comprehensive reviews exist on protein-targeted FBDD [19,41] and on RNA-targeted small-molecule discovery more broadly [42,43], a focused discussion of NMR-based screening approaches specifically applied to RNA targets is still lacking. In this review, we describe the key stages involved in identifying RNA-targeted small molecules using NMR spectroscopy, including fragment library preparation, selecting appropriate NMR screening methods, hit validation and prioritization, fragment optimization, and RNA–ligand complex modeling. We also discuss representative case studies and highlight both the opportunities and challenges of applying NMR-based FBDD to RNA targets.

## 2. Pre-Screening Preparation for RNA-Targeted Fragment Screening

### 2.1. RNA Sample Preparation

**Figure 1:**
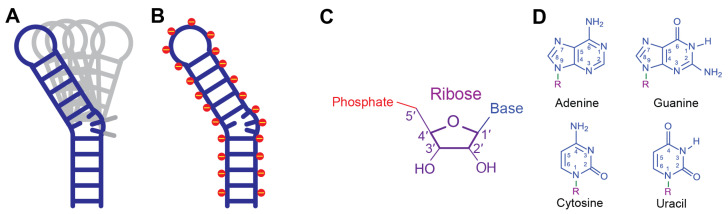
Chemical nature of RNA. Cartoon representation of RNA flexibility (**A**) and its negatively charged backbone (**B**). Schematic of RNA ribose (**C**) and nucleobase (**D**) structures.

Compared to proteins, RNA molecules are often highly conformationally flexible, possess limited chemical diversity, and feature a uniformly negatively charged phosphate backbone, all of which make it challenging to find pockets for small molecule binding (Figure 1). Identifying ligandable RNA structural motifs therefore remains a major bottleneck in RNA-targeted drug discovery [27], despite the great progress that has been made in this field [44,45,46]. Once a suitable RNA construct that contains the potential ligandable pocket has been identified through computational, biochemical, or biophysical approaches, the RNA sample is synthesized either chemically or enzymatically.

In vitro transcription using T7 RNA polymerase remains the most widely used method for producing RNA for NMR studies. This approach enables the generation of milligram quantities of RNA and is compatible with a broad range of isotopically labeled ribonucleotides (rNTPs), including uniformly ^15^N- and ^13^C/^15^N-labeled rNTPs, as well as position-specific ^13^C-labeled rNTPs, which are essential for advanced RNA NMR experiments [15,47,48]. Crude in vitro transcripts are not immediately suitable for screening due to impurities, including residual DNA templates, unincorporated rNTPs, abortive RNA transcripts, and buffer components. These contaminations can be removed by denaturing polyacrylamide gel electrophoresis (PAGE) or by chromatographic techniques such as anion exchange liquid chromatography under high temperatures (85–90 °C) [49,50].

Purified RNA must then be refolded into its biologically relevant conformation. The refolding process, which usually requires heating of the RNA to melt any misfolded conformation and then re-cooling at a specified rate to enable proper folding, often requires extensive construct-specific optimization. This is particularly true for RNAs containing complex tertiary elements such as three-way junctions, G-quadruplex (G4), pseudoknots, or base triples. Improper folding can lead to conformational heterogeneity, which complicates screening and downstream-binding site characterization.

### 2.2. Fragment Library Construction

Fragment libraries differ fundamentally from traditional HTS libraries in that they contain compounds with lower molecular weights but cover a large chemical space. The small size of fragments allows them to explore binding sites more efficiently and to avoid steric clashes that often limit larger compounds [51]. Relatively small libraries, usually in the order of 500 to 3000 compounds, can effectively sample broad chemical space, making fragment screening experimentally tractable. Moreover, the relaxed affinity requirements of fragment screening allow the detection of up to millimolar binders, which would be missed by most HTS activity-based assays.

The “rule of three”, originally proposed by the Astex group over two decades ago, provides a useful guideline for fragment library design [molecular weight < 300; hydrogen bond donors ≤ 3; hydrogen bond acceptors ≤ 3; ClogP ≤ 3, where CLogP denotes calculated lipophilicity (octanol/water partitioning)] [52]. In practice, additional considerations such as solubility at high concentrations (≥1 mM aqueous solubility), chemical stability, and synthetic tractability are equally important. Once suitable fragments are identified, they can be elaborated through merging, linking, or growing strategies to generate higher-affinity lead compounds.

Although numerous commercial suppliers offer fragment libraries, most have been tailor-made for screening against proteins. With an understanding of the nature of RNA–small molecule interaction [53,54,55,56], several RNA-biased or “RNA-privileged” libraries have been developed [57,58,59]. The analyses by the Hargrove group revealed that RNA-binding small molecules preferentially engage in aromatic stacking and hydrogen bonding interactions [54]. Their RNA-targeted bioactive ligand database (R-BIND) further demonstrated that RNA-binding ligands tend to contain a higher proportion of nitrogen atoms, increased ring content, and fewer oxygen atoms relative to protein-targeted compounds [57,60,61,62]. Similarly, the Disney group discovered that benzimidazole scaffold is a favorable scaffold for small molecules to bind RNA [63]. Many RNA-preferring chemical motifs and scaffolds are already present within existing commercial fragment collections, facilitating their repurposing for RNA screening.

### 2.3. Fragment Pooling

Fragment pooling is commonly carried out to increase the throughput of NMR screening experiments [64]. In this strategy, mixtures containing 5–10 fragments are screened simultaneously, substantially reducing the number of experiments required. Making these pools is a critical step for the following screening. Fragments within the same pool should be neither too hydrophobic nor too hydrophilic, and they should have discrete scaffolds from each other with minimal overlapping ^1^H NMR peaks, allowing each fragment to be assessed distinctly by its fingerprint peaks. Cluster analysis can be used to categorize these fragments based on chemical similarities, after which pools are assembled by selecting one fragment from each cluster to ensure a lesser chance of spectral overlap. It is essential that these mixtures are physicochemically compatible and chemically inert, ensuring they remain stable across various analytical and biophysical platforms. After the mixtures are generated, a crucial quality control step is to examine them through NMR in the absence of RNA to confirm that no inter-fragment interactions occur, which would otherwise generate false positives. This is confirmed by taking a baseline spectrum of the mixture first and then comparing them with the individual spectrum of each fragment. Commercial software such as ICM Chemist, ChemDraw, and Mnova, as well as computer-aided scripts, can be utilized in cluster analysis and designing fragment pools taken from a library that has minimized NMR signal overlap [65,66].

^19^F NMR offers a particularly powerful alternative to conventional ^1^H-based screening. The ^19^F nucleus spans a chemical shift range of up to ~800 ppm compared to the ~10 ppm range of ^1^H. ^19^F also has a large gyromagnetic ratio and near 100% natural abundance, making it nearly as sensitive as ^1^H [67,68]. Thus, pools of 20–30 fluorinated fragments can be screened simultaneously with minimal signal overlap, dramatically increasing throughput [69,70].

### 2.4. Screening Conditions

Prior to screening, RNA samples are exchanged into NMR-compatible buffers at desired concentrations. A typical NMR buffer for RNA samples contains 10–100 mM monovalent salt (sodium or potassium) and ~10 mM phosphate buffer at a slightly acidic pH (e.g., 6.4), at which the NMR spectrum has optimal imino and amino proton signals (Table 1) [15]. Millimolar concentrations of magnesium are often required for the folding of RNA that has complex tertiary structures, such as three-way junction and pseudoknots. For a standard 3 mm NMR screening tube, a sample volume of ~160 μL is required for effective NMR shimming to achieve optimal magnetic field homogeneity across the sample.

Fragments are typically prepared as concentrated stock solutions in deuterated dimethyl sulfoxide (DMSO-*d*_6_). While higher sample concentration generates high signal-to-noise spectra, it is typically recommended to keep the concentration of fragments at 50–200 μM with final DMSO-*d*_6_ less than 5% of the total volume. Fragment-to-RNA ratios between 20:1 and 100:1 are typical, and most ligand-observed NMR experiments can be completed within 5–10 min per sample.

## 3. NMR Screening

### 3.1. Integrity of Fragments and Fragment Pools

Before the screening against RNA, each fragment pool and individual fragment should be examined by NMR to verify compound identity, purity, solubility, and the absence of aggregation or self-association. These ensure if any inter or intra-molecular interactions are detected among fragments within pools, they will not be misinterpreted as false positives during the screening. This step can also be used to evaluate the solubility in the solvents, as insoluble compounds could falsely be identified as non-binders. Errors in pooling or sample preparation can often be detected at this stage, preventing any false positives or false negatives during subsequent screening.

### 3.2. The Integrity of RNA

Throughout the screening process, RNA must remain properly folded into the desired conformation. The folding of RNA can be monitored through one-dimensional (1D) imino ^1^H NMR spectra at various concentrations and time points. This NMR data not only provides information on the secondary structure of the RNA, but also can reveal if the RNA aggregates or precipitates in the NMR buffer condition over time. For example, as the fragment solvent, DMSO, can cause RNA degradation and unfolding [71], the final concentration should be maintained below 5% (*v*/*v*). While DMSO at these molar concentrations can subtly perturb RNA through non-specific hydrophobic stacking interaction [71], it lacks the structural complexity to serve as a competitive fragment at the μM levels used for screening. It is therefore critical to collect reference spectra following DMSO addition to confirm that the structured RNA maintains its native fold at the working concentration. Given that screening campaigns often span several days, the stability of RNA over extended periods (a period of at least 96 h) should be evaluated. These evaluations are highly beneficial in eliminating binding to non-authentic RNA aggregates or impurities and reducing false positives in the following steps.

### 3.3. Ligand-Observed Binding Assay

**Figure 2:**
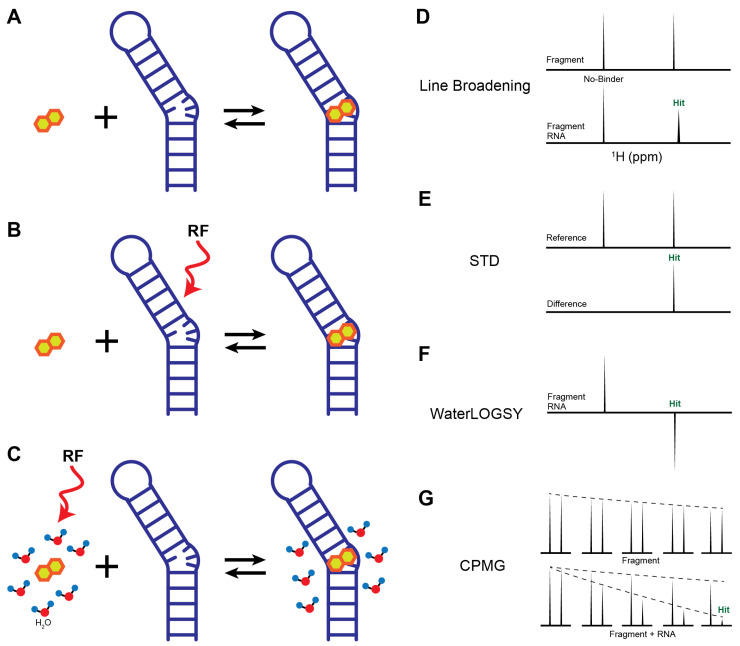
Ligand-based fragment screening methods. (**A**–**C**) Schematic representation of (**A**) line broadening, (**B**) STD, and (**C**) WaterLOGSY. RF is the radio frequency pulse. (**D**–**G**) Cartoon representation of the ligand-based NMR spectra.

Fragment-based screening can be fulfilled by either the ligand-observed or the RNA-observed NMR binding assays (Table 2). Ligand-observed NMR methods offer several advantages: no isotopic labeling requirement, no practical limits on RNA size, and relatively small amounts of RNA sample required, making them cost-effective and high-throughput [11]. Compared to other screening techniques, NMR is particularly sensitive to weak binding interactions, allowing detection of ligands with dissociation constants (K_D_) in the μM–mM range [17,72]. Here, we summarize the most commonly used methods for identifying RNA-targeted small molecules (Figure 2).

1. **Line Broadening**: Line broadening experiments directly compare ligand peak shape, width, or intensity in the absence and presence of RNA (Figure 2A). If interactions occur, the influence of a large RNA molecule on a ligand causes the peak to shift or broaden (Figure 2D). This change can be considered binding and allow the ligands to be identified as initial hits. The extent of line broadening often correlates qualitatively with binding strength and can be used to prioritize hits [73,74]. Therefore, the absence of chemical shift perturbation (CSP) or peak intensity reduction indicates a lack of binding event between RNA and ligands.
2. **Saturation Transfer Difference (STD)**: STD is one of the most widely used ligand-observed techniques in drug discovery and has been successfully applied to RNA targets [75,76,77]. In an STD experiment, RNA resonances (e.g., imino or ribose protons) are selectively saturated through a radio frequency pulse, and then the saturation is rapidly transferred to the entire molecule through spin diffusion (Figure 2B) [78,79]. If binding occurs between RNA and ligand, their proximity causes nuclear Overhauser effect (NOE) transfer from the saturated RNA to the binding site of the ligand. The fast exchange between bound and free ligands results in a reduction in the bulk magnetization of the ligand, while any ligands that do not bind with RNA are minimally affected. However, for high-affinity ligands in the slow exchange regime, the STD effect is markedly reduced or lost. This occurs because the ligands remain trapped in the binding pocket for a duration longer than the saturation period, preventing the accumulation of a detectable signal in the bulk free ligand population. In the optimal exchange regime (typically K_D_ is in the range of μM to mM), the difference in peak intensities between spectra collected with and without saturation only shows the proton signals from the ligand that binds with the RNA (Figure 2E). The relevant reduction in peak intensity also gives additional details on the distance of that proton to the targets as protons that are in closest contact with the RNA surface exhibit the most intense STD signals. Thus, STD not only confirms binding but also provides epitope mapping information on ligand protons proximal to the RNA surface.
3. **Water-Ligand Observed via Gradient Spectroscopy (WaterLOGSY)**: WaterLOGSY is another popular NMR screening method that detects ligand-RNA interactions through water-mediated NOEs [80,81]. In a WaterLOGSY experiment, bulk water magnetization is selectively excited and subsequently transferred to ligands in solution (Figure 2C) [80]. Upon binding, water magnetization is further transferred to RNA-bound ligands via intermolecular NOEs. The tumbling rate of free ligands is much faster than the rate of RNA-bound ligands. Due to the slower tumbling rate of RNA–ligand complex, the NOE sign of bound ligands is inverted relative to that of free ligands, resulting in opposite signal phases in the spectrum (Figure 2F) [80]. Similar to STD experiments, WaterLOGSY is insensitive to the tight binding events, but is often more sensitive than STD for detecting weak fragment binding [82]. As a water-mediated technique, WaterLOGSY is not suitable for the hydrophobic pockets where water is absent, but it can provide useful information on solvent-exposed regions of ligands [21]. Given the intrinsically hydrophilic nature of RNA surfaces, WaterLOGSY is particularly well suited for RNA-targeted fragment screening [83].
4. **Carr–Purcell–Meiboom–Gill (CPMG)**: CPMG probes binding interactions by exploiting chemical exchange and differences in transverse relaxation rates between the free and bound states of a ligand. Free ligands typically exhibit long transverse relaxation times (*T_2_*) due to rapid molecular tumbling, whereas ligands bound to RNA experience substantially shortened *T_2_* values as a consequence of the slower tumbling of the RNA–ligand complex. As a result, NMR signals from RNA-bound ligands decay more rapidly than those from free ligands under a CPMG pulse train, providing a sensitive readout of binding (Figure 2G). In addition to ^1^H detection, CPMG experiments can be implemented using other nuclei, such as ^19^F, which offers high sensitivity, a wide chemical shift dispersion, and minimal background signals in biological samples. ^19^F-CPMG has been successfully applied to identify fluorinated fragments that bind selectively to telomeric repeat-containing RNA (TERRA) G4 and SARS-CoV2 RNAs, highlighting its utility for RNA-targeted fragment screening [84,85].

### 3.4. RNA-Observed Binding Assay

RNA-observed methods directly monitor changes in RNA resonances upon ligand binding. These methods typically require larger quantities of RNA than the ligand-observed assays, but they can provide binding information on a base pair, residue, or atomic level within RNA molecules (Table 2). Due to the rich information but relatively low throughput, these experiments best serve as a tool to validate the primary hits and map binding sites.

**Figure 3:**
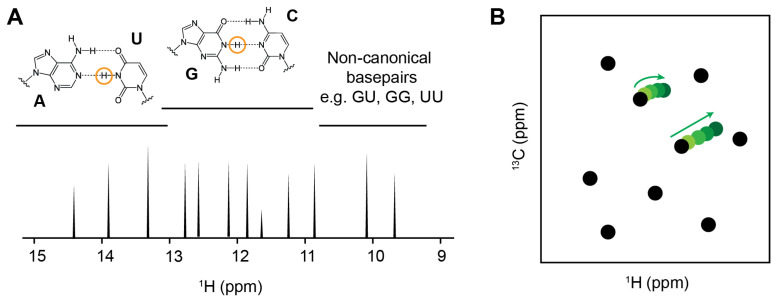
RNA-based NMR methods. (**A**) RNA imino proton spectrum. Typical imino chemical shift ranges are indicated. (**B**) Cartoon representation of 2D ^1^H-^13^C HSQC spectrum. Selected crosspeaks with chemical shift changes are highlighted.

1. **1D Imino Proton Spectroscopy**: Imino proton spectroscopy, which detects the H1 proton of guanosine and the H3 proton of uridine, provides a sensitive means to monitor any binding at the base-pair level with the advantage of no isotopic labeling requirement [84]. Imino proton signals arise from only the hydrogen-bonded base pairs and typically resonate in a well-dispersed chemical shift region between ~9 to 15 ppm (Figure 3A). This is largely away from the overlap with other proton peaks that come from small-molecule fragments [15]. With the assignment of the imino peaks, which is commonly achieved through routine 2D imino ^1^H-^1^H nuclear Overhauser effect spectroscopy (NOESY), the binding site on the RNA can be mapped based on the ligand-induced CSPs or intensity changes. However, since imino proton peaks come from stable hydrogen bonding within the base pairs, unstructured or dynamic elements, such as loops, bulges, and single-stranded regions, are absent in the imino proton spectra. Therefore, 1D imino proton experiment may fail to detect ligand interactions that occur primarily within flexible or non-base-paired RNA regions.
2. **Total Correlation Spectroscopy (TOCSY)**: TOCSY is a common RNA NMR experiment that does not require isotopic labeling and fits well with monitoring RNA folding and ligand-induced conformational changes. Through scalar coupling networks, TOCSY detects through-bond correlations between H5 and H6 protons of pyrimidine residues (uridine and cytidine) with high sensitivity [15]. These crosspeaks are in a distinct chemical shift range (H5 protons generally resonate between 5.0 and 6.0 ppm, while H6 protons resonate from 7.0 to 8.5 ppm) and are typically well resolved in folded RNAs, which allow detection of subtle interactions related to pyrimidines at the residue level [86,87]. Complementary to the imino proton spectroscopy, TOCSY can observe interactions that involve pyrimidines located in unstructured or dynamic regions such as loops and bulges, which are common RNA structural motifs and frequently serves as small-molecule binding pockets. Notably, the Varani group has recently employed TOCSY-based screening to identify fragment hits that bind the unstructured apical loop of the precursor microRNA-21 (pre-miR-21), demonstrating the utility of this approach for probing flexible RNA elements [74].
3. **Heteronuclear Single Quantum Correlation Spectroscopy (HSQC)**: HSQC is a widely used in protein-targeted drug discovery to monitor ligand interactions via amide proton resonances. In RNA, however, aromatic (H2, H5, H6, and H8), ribose (H1′, H2′, H3′, H4′, H5′, and H5″), and imino (H1 and H3) protons could be severely overlapped in 1D proton spectra along with the increasing of their size or structural complexity. This overlap can be effectively resolved with the additional heteronuclear dimension, making high-resolution analysis of RNA–ligand interactions possible (Figure 3B) [15]. Although HSQC experiments typically require isotopically labeled RNA, the spectra are able to provide valuable binding information at the atomic level. CSP or titration experiments using ^1^H-^13^C HSQC spectra allow the characterization of ligand interactions involving RNA bases and ribose moieties, while ^1^H-^31^P HSQC experiments are sensitive to perturbations of the phosphate backbone. With the recent development of new pulse sequences, such as SOFAST and other fast-pulsing experiments, together with nonuniform sampling strategies, HSQC-based approaches, in some cases, have the ability to directly observe non-labeled RNA [88,89].

## 4. Hit Ranking and Optimization

Before ranking and optimizing primary hits, it is critical to validate and mitigate false positives, which are a common challenge in RNA-targeted fragment screening. These artifacts often arise from fragment aggregation or non-specific electrostatic interactions with the polyanionic RNA backbone. To ensure hit authenticity, several mitigation strategies are typically employed: (1) the addition of low concentrations of non-ionic detergents (e.g., 0.01% Triton X-100) to disrupt small-molecule aggregates; (2) performing dose–response NMR titrations to ensure binding is saturable; and (3) cross-validating hits using at least two independent NMR assays, such as combining a ligand-observed STD experiment with an RNA-observed imino shift assay.

After hit validation, the focus shifts to ranking and prioritizing hits for downstream characterization. Since the initial fragment screening typically does not obtain quantitative binding affinities and full titration experiments to measure K_D_ are labor intensive, efficient ranking strategies are required for the fragment screening campaign. To streamline this, the Varani group recently introduced a practical hit ranking approach based on the ratio of peak intensities corresponding to the bound and free ligand states, enabling rapid estimation of fractional bound occupancy [74]. Empirically, fragments with a fractional bound occupancy greater than 0.4 were shown to have a higher likelihood of being confirmed as true RNA binders. Alternatively, a complementary prioritization strategy is to rank hits based on consistency across multiple NMR screening assays. The fragments that exhibit consistent binding signatures in several screening methods (e.g., line broadening, STD, WaterLOGSY, and CPMG) are prioritized over those detected by a single method [71]. For fluorinated fragments, cross-validation using both ^1^H and ^19^F-detected line broadening and CPMG experiments provides an additional layer of confidence and helps minimize false-positive hits [85].

Validated high-priority fragments then enter structure-guided medicinal chemistry cycles [90]. Once key functional groups responsible for RNA binding and desired biological activity are identified, fragments can be grown through systematic chemical modification within a multi-parameter optimization framework to affinity, solubility, stability, and drug-like properties. When two fragment hits share a common scaffold, they can be merged to combine their favorable interactions and boost potency [91]. Additionally, two validated hits that bind distinct sites can be linked through an appropriate chemical linker, potentially yielding substantial gains in affinity and specificity through additive binding energies [92]. Linker design typically benefits from high-resolution structural information to make sure proper spatial orientation and minimal entropic penalty. Throughout the optimization process, both ligand- and RNA-observed NMR experiments play central roles in guiding SAR and validating binding modes [93,94].

## 5. RNA–Ligand Structural Modeling

Identifying the binding mode of small molecules to RNA is a critical step in FBDD, as it builds the foundation for structure-guided hit-to-lead optimization. X-ray crystallization can provide atomic resolution views of RNA–ligand interfaces, but it remains challenging to crystallize RNA or RNA–ligand complex, particularly for RNAs containing flexible and dynamic regions. Despite the fast advances of cryo-EM over the past decade which enables RNA structure determination at near-atomic or atomic resolution, this technique remains largely restricted to large, well-structured RNAs [95,96,97,98].

**Figure 4:**
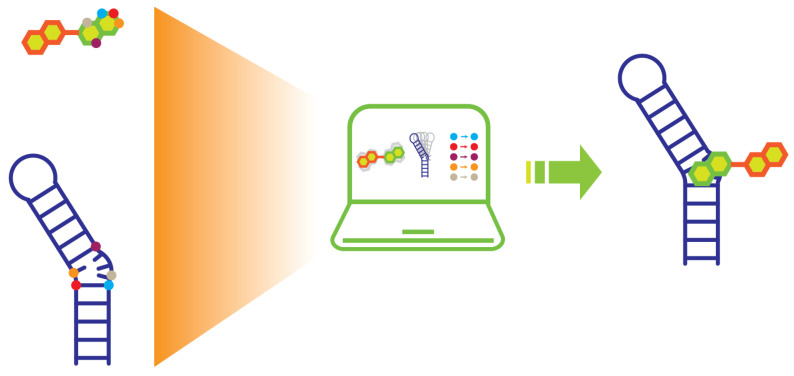
Cartoon representation of computational modeling workflow of an RNA–small molecule complex. The binding interfaces on small molecule and RNA obtained from NMR binding assay are indicated with colored dots. The dots that have the same color indicate that the two hydrogen atoms are close to each other within 5 Å.

NMR spectroscopy offers a versatile alternative for generating atomic-level structures of RNA–ligand complexes and is especially well-suited for structure-guided hit-to-lead evolution. Binding information obtained during NMR-based screening and validation experiments can be directly integrated into structural model building (Figure 4). NMR is a well-established biophysical tool for determining the high-resolution structure of RNA and RNA–ligand complexes in solution [47,99,100,101]. The NOESY experiment forms the cornerstone of NMR-based structural determination by detecting through-space correlations between protons separated by distances typically less than 5 Å. The distance information on protons from various locations across the RNA and between the RNA and ligand are used as restraints to define its three-dimensional structure.

The conventional RNA structure determination through NMR requires extensive data collection and peak assignment, which can be time-consuming. Recent advances have demonstrated that reliable RNA structural models can be calculated using sparse distance restraints obtained from imino proton resonances, particularly when combined with molecular dynamics simulations [102]. However, this sparsity can result in an under-determined structural system where the global fold is well-defined, but the precise orientation of specific nucleotides or bases remains uncertain. For RNA–ligand complexes, the general workflow has shifted toward an integrative approach: (1) obtaining intermolecular NOEs, which can be collected via filtered/edited NOESY experiments [103], to provide critical restraints for defining the binding interface and ligand orientation (Figure 4); (2) utilizing computational docking platforms like HADDOCK to integrate these NOEs with the CSP data obtained from NMR binding assays for rigid body or semi-flexible docking [104]; (3) applying simulated annealing or molecular dynamics, such as Xplor-NIH [105] and Amber [106], to optimize local geometry and solvent interactions; (4) identifying the most energetically favorable models that remain consistent with all experimental data. These models can be further refined by advanced NMR experiments, including SALMON (solvent accessibility, ligand binding, and mapping of ligand orientation by NMR spectroscopy) [107] and DEEP-STD (differential epitope mapping by STD NMR) [75], to characterize detailed RNA–ligand interactions.

## 6. Case Studies in NMR-Based RNA Fragment Screenings

**Figure 5:**
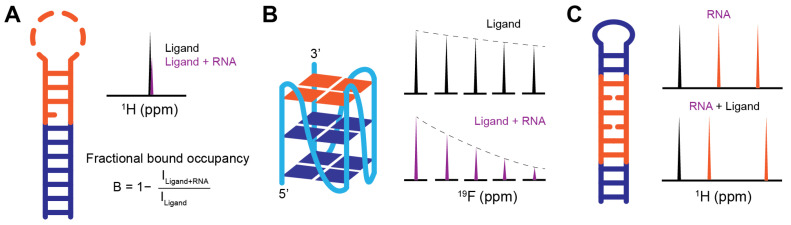
Case studies of fragment screen against pre-miR-21, TERRA G4, and DM1 CUG hairpin RNA. The orange regions in the secondary structure are the proposed binding site. (**A**) 1D ^1^H spectra of line broadening. In the equation, B is the fractional bound occupancy peak intensity represented by bound (I_Ligand+RNA_) and free (I_Ligand_) ligand. (**B**) ^19^F CPMG signal of ligand with or without RNA. (**C**) 1D ^1^H spectra indicate that the ligand causes chemical shift perturbation at the CUG region (orange) in the presence of ligand.

To illustrate the practical application of NMR-based fragment screening across structurally and functional diverse RNA targets, we highlight three representative case studies (Figure 5). These examples demonstrate distinct screening strategies, including ligand-observed, RNA-observed, and ^19^F-based NMR approaches, and highlight how NMR can reveal not only fragment binding but also RNA conformational remodeling to biological function.

### 6.1. Discovery of Fragment Binders to Pre-miR-21

MicroRNA-21 is a highly conserved small non-coding RNA that plays a central role in regulating cell survival, proliferation, and tumor suppression [108]. Pre-miR-21 features an apical loop structural motif that is essential for Dicer-mediated processing into mature miR-21 (Figure 5A). Consequently, this loop represents a high-priority target for anti-cancer therapeutics aimed at inhibiting miRNA maturation.

Approach: The Varani group implemented a systematic NMR-based screening campaign specifically targeting the pre-miR-21 apical loop [74]. A Maybridge fragment library consisting of 420 compounds was organized into 54 mixtures (5–8 ligands per pool) and screened using ligand-observed line broadening experiments in a deuterated buffer. Because the apical loop is highly dynamic and lacks stable hydrogen-bonded base pairs, it does not produce detectable imino signals. To overcome this, the team employed RNA-observed TOCSY experiments, monitoring the aromatic protons of pyrimidine, to directly observe CSPs within the loop region upon fragment binding.

Results: 17 hits (hit rate 4%) were identified through the first round of screening. A significant contribution of this study was the introduction of a quantitative prioritization strategy using fractional bound occupancy, which was estimated based on the ratio of peak intensities between the bound and free states of the ligands (Figure 5A). Fragments with a value greater than 0.4 were prioritized for further study. Several fragments selectively perturbed TOCSY cross-peaks of pyrimidines within the pre-miR-21 loop, and subsequent NOESY experiments provided definitive evidence of binding through the detection of intermolecular NOEs. This study highlighted the power of combining ligand-observed screening with residue-specific RNA-observed validation for highly dynamic motifs.

### 6.2. ^19^F NMR Screening Against TERRA G-Quadruplexes

TERRA is a long non-coding RNA transcribed from telomere repeats that is essential for maintaining chromosomal integrity [109,110]. These G-rich transcripts tend to fold into RNA G4 structures (Figure 5B) [111], which present expansive G-tetrad surfaces and unique topologies that are highly amenable to small-molecule recognition. While G4 structures can exhibit significant polymorphism, human telomeric RNA is well-established in the literature to favor a homogenous, propeller-like parallel topology in the presence of K^+^ ions [112,113].

Approach: To exploit the high sensitivity and wide chemical shift dispersion of fluorine, researchers utilized a ^19^F NMR platform to screen a library of 355 fluorinated fragments containing either trifluoromethyl (CF_3_) or monofluoride (CF) groups [84]. The authors first employed CD and NMR spectroscopy to confirm that their TERRA construct adopted a homogenous G4 topology in K^+^-rich buffer. During the primary screening, fragments were evaluated in pools of eight using ^19^F CPMG experiments (Figure 5B). The sensitive ^19^F signals allowed for the clean detection of binding-induced relaxation changes, even with RNA concentrations as low as 1 μM, significantly reducing the material requirements for the target RNA.

Results: The primary screening identified 20 initial hits, representing a hit rate of approximately 5.6%. From this group, seven fragments were prioritized based on their solubility. Secondary validation using both additional ^19^F CPMG and ^1^H-based STD confirmed that the majority were authentic binders. To assess selectivity, the authors evaluated hit binding against tRNA, a DNA duplex, and a DNA G4. The results confirmed that these ligands selectively recognize the specific topologies of propeller-like parallel RNA G4s over other nucleic acid architectures. This case study underscores that ^19^F NMR is an exceptionally sensitive approach for detecting weak interactions with a low false-positive rate, making it ideal for the discovery of selective RNA binders.

### 6.3. Targeting the Myotonic Dystrophy CUG Repeats

Myotonic dystrophy type 1 (DM1) is an inherited multisystem genetic disorder caused by the expansion of CUG triplet repeats in the DMPK gene [114]. These expanded repeats fold into stable hairpins that sequester the alternative splicing regulator Muscleblind-like 1 (MBNL1) (Figure 5C), leading to toxic gain-of-function effects and widespread splicing dysfunction [115]. Disrupting the CUG–MBNL1 interaction with small molecules is a promising therapeutic strategy for restoring normal splicing patterns.

Approach: To identify an RNA-targeted covalent binder, the Disney lab developed a fragment-based covalent drug discovery approach, employing a library of 187 “fully functionalized” fragments containing a photoreactive diazirine moiety and an alkyne tag [116]. To increase throughput, the primary screening was performed using a fluorescence-based binding assay. The hits were subsequently validated and optimized using the fragments’ non-covalent cores through both ligand- and RNA-observed NMR experiments. Once the core scaffolds were optimized, the covalent warhead was re-integrated to form the final reactive probes.

Results: The initial fluorescence screen yielded 14 hits, which were then prioritized via ligand-observed NMR to confirm the scaffolds bind specifically at the CUG region of the RNA (Figure 5C). RNA-observed imino proton spectroscopy was extensively utilized during the hit optimization phase to guide fragment linking and ensure it is on target. This process led to the development of a dimeric compound that exhibited high selectivity and effective covalent modification of the CUG repeats. This study highlights how NMR-based fragment screening, when integrated with high-throughput biochemical assays, can streamline the development of high-affinity binders and even proximity-induced covalent ligands for challenging RNA targets.

## 7. Summary and Future Perspectives

The growing recognition of disease-relevant RNAs has stimulated increasing interest in the development of RNA-targeted small-molecule therapeutics (Table 3). The 2020 FDA approval of the first RNA-targeted small-molecule drug, risdiplam, for the treatment of spinal muscular atrophy provides compelling validation that structured RNAs can serve as druggable targets [117,118]. Compared with proteins, RNA exhibits distinct biophysical characteristics, including intrinsic conformational flexibility and a highly negatively charged surface, which require tailored discovery strategies and screening methodologies. Continued expansion of RNA-privileged small molecule libraries, together with the rapidly increasing number of experimentally determined RNA and RNA–ligand complex structures, is expected to elucidate fundamental principles governing RNA recognition and remodeling by small molecules. These advances will, in turn, inform the rational design of fragment libraries increasingly optimized for RNA targeting [57,99,119,120,121,122,123].

Relative to HTS campaigns against classical protein targets, RNA-targeted FBDD remains a comparatively less mature field. Nevertheless, a wide range of screening approaches has already been explored, with NMR spectroscopy emerging as a particularly powerful and versatile technique. A particularly promising direction is the integration of fragment-based covalent drug discovery. NMR spectroscopy is uniquely positioned to guide this process, as it can be used to optimize the non-covalent recognition core before engaging the covalent warhead, thereby promoting high specificity and reducing off-target effects in the cellular environment. Ongoing development of RNA-specific NMR methodologies, such as ^19^F-based screening strategies and improved RNA-observed assays, will be critical for increasing throughput and reducing false-positive rates. Moreover, combination of NMR with complementary biophysical and computational techniques, including X-ray crystallography, SPR, cryo-EM, computational modeling, and machine learning, will enable more efficient identification, validation, and optimization of authentic RNA binders [133,134,135].

NMR has clearly established itself as a mainstream approach in protein-targeted drug discovery [11,128,136,137]. Its unique strengths in detecting weak interactions and providing site-specific structural information simultaneously during screening position it to play an increasingly central role in RNA-targeted FBDD. As methodological innovations continue and our understanding of RNA–small-molecule interactions deepen, NMR-based FBDD will significantly expand the landscape of druggable RNA targets and accelerate the development of RNA-directed therapeutics.

## Figures and Tables

**Table 1:** Recommended experimental parameters for RNA NMR fragment screening.

Parameter	Typical Range	Notes/Best Practice
RNA concentration	1–100 μM	Lower for ligand-observed; higher for RNA-observed
Fragment concentration	50–200 μM	Avoid aggregation; confirm solubility
Fragment:RNA ratio	20:1–100:1	Keep detectable bound fraction
DMSO (%)	≤5%	Check if DMSO concentration affects RNA integrity
Mg^2+^	0–5 mM	Required for tertiary structure
Buffer	10 mM phosphate, pH~6.4	Optimizes imino visibility
Temperature	278–298 K	Balance stability vs. dynamics

**Table 2:** Comparison of NMR screening methods for RNA-targeted fragment discovery.

Observation Type	NMR Method	RNA Size	Sensitivity (K_D_ Range)	Labeling Required?	RNA Amount Required	Strengths	Key Limitations
Ligand	LB	Any	μM–mM	No	Low (~2–20 μM)	Simple, fast, low sample	False positives from aggregation
	STD	Any	μM–mM	No	Low (~2–20 μM)	Epitope mapping	Insensitive to tight binders
	WaterLOGSY	Any	μM–mM	No	Low (~2–20 μM)	High sensitivity	Requires solvent-exposed sites
	CPMG	Any	μM–mM	No	Low (~2–20 μM)	High sensitivity	Sensitive to exchange regime
	^19^F NMR	Any	μM–mM	No	Very low (~0.5–10 μM)	Clean spectra	Requires fluorinated library
RNA	1D imino	≤~100 nt	nM–mM	No	Moderate (~10–50 μM)	Base-pair level mapping	Misses unpaired regions
	TOCSY	≤~80 nt	nM–mM	No	Moderate (>50 μM)	Loop/bulge sensitivity	Pyrimidine-only
	HSQC	≤~70 nt *	nM–mM	Yes	High (>50 μM)	Atomic resolution	Sample preparation cost

* ^13^C or ^15^N isotopically labeled RNA.

**Table 3:** Representative NMR-based fragment screening studies targeting RNA targets.

RNA Target	Disease/Application	Fragment Library	NMR Methods	Hit Ranking and Triage Approach	Reference
Bacterial ribosomal A-site model RNA	Antibiotics (translation inhibition)	RNA-directed fragment library (102 compounds)	WaterLOGSY and ^1^H CPMG	5 hits were confirmed by RNA-observed imino proton	Bodoor et al., J. Med. Chem. (2009) [124]
Thiamine pyrophosphate riboswitch thiM	Riboswitch	Fragment library of 1300 compounds	WaterLOGSY and ^1^H CPMG	17 primary hits; further validated by other biophysics assay: equilibrium dialysis and ITC	Cressina et al., Chem. Sci. (2011) [125]
HIV-1 TAR RNA	Antiviral (Tat–TAR interaction)	Maybridge “Rule of 3” collection (250 compounds)	STD, interligand NOEs (ILOE)	20 primary hits; ranked by STD signal intensity; 6 hits were confirmed by ILOE	Davidson et al., Chem. Biol. (2011) [77]
TERRA G-quadruplex	Cancer/telomere biology	Fluorinated fragment library (355 compounds)	^19^F CPMG	20 primary hits; 6 were confirmed by STD and RNA-observed imino proton	Garavís et al., ACS Chem. Biol. (2014) [84]
Influenza A promoter RNA	Antiviral (influenza)	Fragment library of 4279 compounds	1D imino proton	7 hits; the one that has the most dramatic CSP was advanced to structural determination	Lee et al., Chem. Commun. (2014) [126]
M. tuberculosis rRNA PTC/hairpin 91	Tuberculosis antibiotics	Maybridge “Rule of 3” collection (1000 compounds)	^1^H CPMG	9 primary hits; hit expansion with virtual library; ranked on the basis of the binding energy	Tam et al., Chem. Sci. (2019) [127]
14 structured RNAs (incl. riboswitches)	Multi-disease	Fluorinated fragment library (102 compounds)	^19^F CPMG	Hit rate up to 26% for some targets; ranked by the CPMG signal	Binas et al., ChemBioChem (2021) [128]
Pre-miR-21 apical loop	Cancer/miRNA dysregulation	Maybridge “Rule of 3” collection (420 compounds)	Line broadening	17 primary hits; ranked by fractional bound occupancy; 4 hits were confirmed by NOESY	Shortridge and Varani, ACS Med. Chem. Lett. (2021) [74]
15 conserved SARS-CoV-2 RNA elements	Antiviral (COVID-19)	The DSI-poised library (768 compounds)	CSP, WaterLOGSY, line broadening, ^1^H CPMG	69 primary hits across targets; ranked by consistent binding across multiple methods	Sreeramulu et al., Angew. Chem. Int. Ed. (2021) [33]
SARS-CoV-2 5′UTR stem loops	Antiviral (COVID-19)	RNA-dedicated fluorinated DRTL-F library (49 compounds) and non-RNA-dedicated DSI-PL (768 compounds)	Parallel ^1^H/^19^F CSP and ^19^F CPMG	10 primary hits from DRTL-F library and 5 primary hits from DSI-PL library.	Hymon et al., RSC Med. Chem. (2024) [85]
Myotonic dystrophy type 1 CUG repeats	Muscular dystrophy	Fully functionalized fragment library (187 compounds)	CSP/line broadening, WaterLOGSY, ^1^H CPMG	14 primary hits from primary fluorescent binding screening; 4 were confirmed by NMR assay; 1 was picked based on consistent binding across multiple NMR methods	Jia et al., ACS Chem. Biol. (2025) [116]
Theophylline aptamer	Riboswitch	Fragment library (1975 compounds)	1D imino proton	28 primary hits; 4 hits were confirmed by SPR	Kwai et al., ACS Chem. Biol. (2025) [129]
Human cytoplasmic A-site and the S. cerevisiae tRNA anticodon stem loop with and without modification	Translation	RNA-optimized fluorinated fragment library + 2 non-optimized libraries (149 to 529 compounds depend on the targets)	WaterLOGSY and ^19^F CPMG	24, 31, and 20 primary hits against the respective targets from ^19^F screening. Secondary WaterLOGSY screening verifies a few positive binders	Lundquist et al., SLAS Discov. (2025) [130]
Riboswitches (FMN, SAM-I, and TPP)	Riboswitch	Fragment library (651 compounds)	WaterLOGSY, CSP, ^1^H CPMG	35 primary hits from biolayer interferometry; 7 verified by secondary NMR screening were used to confirm the specificity	Panchal et al., RSC Med. Chem. (2025) [131]
SARS-CoV-2 5′UTR stem-loop 1	Antiviral (COVID-19)	Lead-derived library (41 compounds)	CSP, WaterLOGSY, line broadening, ^1^H CPMG, TOCSY	Ranked by consistent binding across multiple methods; confirmed by a counterscreen; further confirmed by TOCSY	Toews et al., JACS (2025) [132]

## Data Availability

No new data were created or analyzed in this study. Data sharing is not applicable to this article.

## Abbreviations

The following abbreviations are used in this manuscript:

1D	one-dimension
CF	monofluoride
CF_3_	trifluoromethyl
CPMG	Carr–Purcell–Meiboom–Gill
Cryo-EM	cryo-electron microscopy
CSP	chemical shift perturbations
DEEP-STD	differential epitope mapping by STD NMR
DMSO-*d*_6_	deuterated dimethyl sulfoxide
FBDD	fragment-based drug discovery
G4	G-quadruplex
HSQC	heteronuclear single quantum correlation spectroscopy
HTS	high-throughput screening
K_D_	dissociation constants
LB	Line broadening
MBNL1	Muscleblind-like 1
NMR	nuclear magnetic resonance
NOE	nuclear Overhauser effect
NOESY	nuclear Overhauser effect spectroscopy
PAGE	polyacrylamide gel electrophoresis
R-BIND	RNA-targeted bioactive ligand database
rNTP	ribonucleotides
SALMON	solvent accessibility, ligand binding, and mapping of ligand orientation by NMR spectroscopy
SAR	structure–activity relationship
SPR	surface plasmon resonance
STD	saturation transfer difference
TERRA	telomeric repeat-containing RNA
TOCSY	total correlation spectroscopy
WaterLOGSY	water-ligand observed via gradient spectroscopy
